# Virtual conversational agents versus online forms: Patient experience and preferences for health data collection

**DOI:** 10.3389/fdgth.2022.954069

**Published:** 2022-10-13

**Authors:** Hiral Soni, Julia Ivanova, Hattie Wilczewski, Alexandra Bailey, Triton Ong, Alexa Narma, Brian E. Bunnell, Brandon M. Welch

**Affiliations:** ^1^ Doxy.me Research, Doxy.me Inc., Rochester, NY, United States; ^2^ Department of Psychiatry and Behavioral Neurosciences, Innovation in Mental Health Lab, University of South Florida, Tampa, FL, United States; ^3^ Department of Public Health Sciences, Medical University of South Carolina, Charleston, SC, United States

**Keywords:** health data collection, patient experience, virtual conversational agents, chatbots, usability

## Abstract

**Objective:**

Virtual conversational agents, or chatbots, have emerged as a novel approach to health data collection. However, research on patient perceptions of chatbots in comparison to traditional online forms is sparse. This study aimed to compare and assess the experience of completing a health assessment using a chatbot vs. an online form.

**Methods:**

A counterbalanced, within-subject experimental design was used with participants recruited *via* Amazon Mechanical Turk (mTurk). Participants completed a standardized health assessment using a chatbot (i.e., Dokbot) and an online form (i.e., REDCap), each followed by usability and experience questionnaires. To address poor data quality and preserve integrity of mTurk responses, we employed a thorough data cleaning process informed by previous literature. Quantitative (descriptive and inferential statistics) and qualitative (thematic analysis and complex coding query) approaches were used for analysis.

**Results:**

A total of 391 participants were recruited, 185 of whom were excluded, resulting in a final sample size of 206 individuals. Most participants (69.9%) preferred the chatbot over the online form. Average Net Promoter Score was higher for the chatbot (NPS = 24) than the online form (NPS = 13) at a statistically significant level. System Usability Scale scores were also higher for the chatbot (i.e. 69.7 vs. 67.7), but this difference was not statistically significant. The chatbot took longer to complete but was perceived as conversational, interactive, and intuitive. The online form received favorable comments for its familiar survey-like interface.

**Conclusion:**

Our findings demonstrate that a chatbot provided superior engagement, intuitiveness, and interactivity despite increased completion time compared to online forms. Knowledge of patient preferences and barriers will inform future design and development of recommendations and best practice for chatbots for healthcare data collection.

## Introduction

Patient-reported health data such as medical history and clinical questionnaires are a vital and routine component of care and research. Patient-reported data give providers a more comprehensive picture of a patient's condition so they can make informed decisions and provide high quality care. In research, patient data are vital to assess progress, make inferences, and obtain meaningful insights to improve health.

Conventional data collection methods such as paper-based or online forms can be time-consuming, unintuitive, and full of jargon (1). The language used in these forms often requires an advanced reading level to understand (1). Further, data collection *via* paper forms may require costly and error-prone human input (2–4). In addition, online forms must comply with privacy regulations, integrate with different tools for efficient data collection and sharing, and provide an effective user experience to ensure high-quality data–free of omissions and inaccuracies (5). Beyond paper or online forms, verbal data collection by clinicians and/or researchers is easier for patients to complete; elicit higher quality information; and generate discrete, accessible data for research (6). However, this approach requires dedicated staff, an option that is not always practical or affordable (7–9).

Virtual conversational agents (i.e., chatbots) have proven to be a promising approach to patient data collection. Chatbots simulate human conversations to provide a data collection experience that is more naturalistic and intuitive than standard forms. Patients have reported positive perceptions of chatbots including ease of use, understandability, and enjoyability (10–15). Some studies have found that chatbots improve user experience by reducing workload and enhancing ease of completing asynchronous surveys at patients’ convenience (16). Providers perceive chatbots as beneficial for administration and organizational tasks and information dissemination (17).

Research on patient experiences with data collection chatbots in comparison to conventional online or web-based forms is sparse. One study comparing family health history collection using a chatbot against an online form found that participants reported higher satisfaction, usefulness, information quality, and interface quality for the chatbot despite overall longer completion time (11). However, participants in this study were recruited from a university campus, were highly educated, and completed the study in a controlled environment with fictional scenarios and data. There is a need to investigate chatbot preferences and optimization with larger, more diverse populations.

The purpose of the present study was to compare patient data-capture experience of chatbots with online forms. The findings of this study will help understand individuals’ experiences and preferences of data collection using a chatbot and will establish recommendations for chatbot development and usability in the future.

## Methods

### Study settings and participants

This online study was completed *via* Amazon Mechanical Turk (mTurk), an online crowd-sourcing platform for remote recruitment (18). We aimed to recruit 400 mTurk workers (individuals registered as potential participants on mTurk) based in the US and ≥18 years of age by using mTurk's worker requirement filtering. After providing informed consent, participants completed the study as described in Section “Study design”. No personally identifiable information was collected. Participants were compensated $2.50 to complete this 20-minute study. This study was designated exempt by the Medical University of South Carolina Institutional Review Board (Pro00082875).

### Study measures

#### Demographics

We collected information on participant characteristics (age, race, ethnicity, education, gender) and previous experience of taking surveys, including commonly used devices to complete the surveys and familiarity with survey taking.

#### System usability scale (SUS)

The SUS includes 10 items that are alternatingly worded positively (e.g., I thought the system was easy to use) and negatively (e.g., I found the system unnecessarily complex)(19). Responses were anchored on a 5-point Likert Scale (i.e., 1 = *Strongly Disagree* to 5 = *Strongly Agree*).

#### Net promoter score (NPS)

The NPS is a one-question measure of customer loyalty and likelihood to recommend a product (How likely are you to recommend 〈tool name〉 as a survey completion tool?) and is considered a gold-standard rating (20).

#### Tool preference

We asked about participants' tool preference (chatbot vs. online form) using a structured question [i.e., Based on your experience today with the two survey tools, which of the following tools would you prefer? (Dokbot; REDCap)] and three open-ended questions regarding likes and dislikes of each tool and tool enhancement suggestions.

#### Assessment completion time

We measured the assessment completion time (in seconds) as the time from when the user was directed to a tool (chatbot or online form) to when the user was directed to the UXEvaluator (Section “Study design”).

### Study design

This study used a counterbalanced, within-subject experimental design to conduct a large-scale, unmoderated study comparing the usability and user experience of completing a health assessment using a chatbot (i.e., Dokbot) and an online form (i.e., REDCap) (11, 21).

Dokbot is a free, secure (compliant with Health Insurance Portability and Accountability Act of 1996), simple chatbot developed to collect healthcare data in an interactive way, mimicking human-to-human interaction. Dokbot is a browser-based application that does not require downloads and is designed with a mobile-first approach, which is particularly important for patients as they are most likely to access the internet through a smartphone (22, 23). Dokbot can be customized with various names, avatars, languages, and personalities appropriate to user characteristics (e.g., age, gender, etc.) and can be integrated within different health information technology (HIT) systems (24).

**Figure 1:**
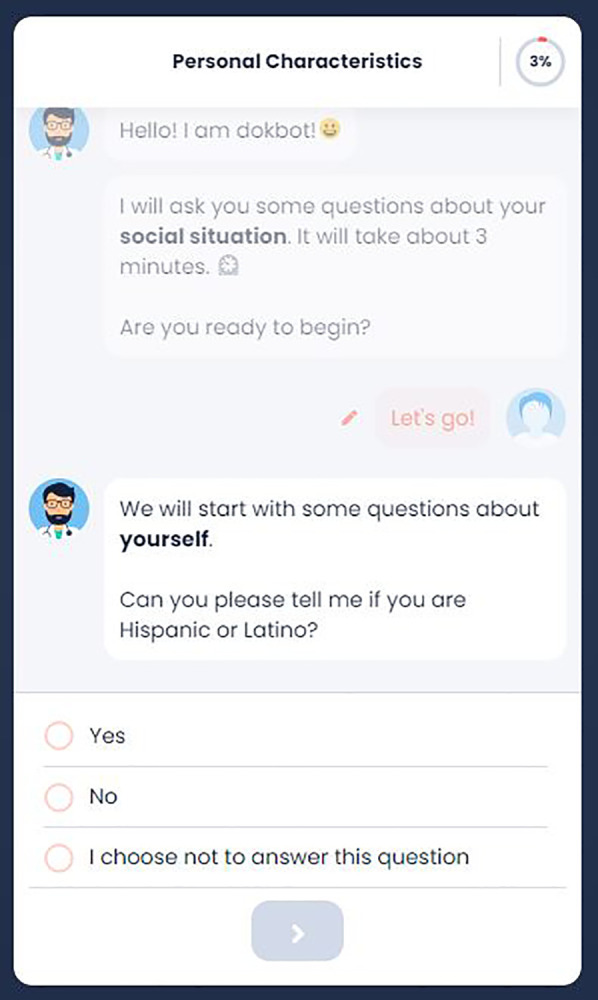
Dokbot interface.

**Figure 2:**
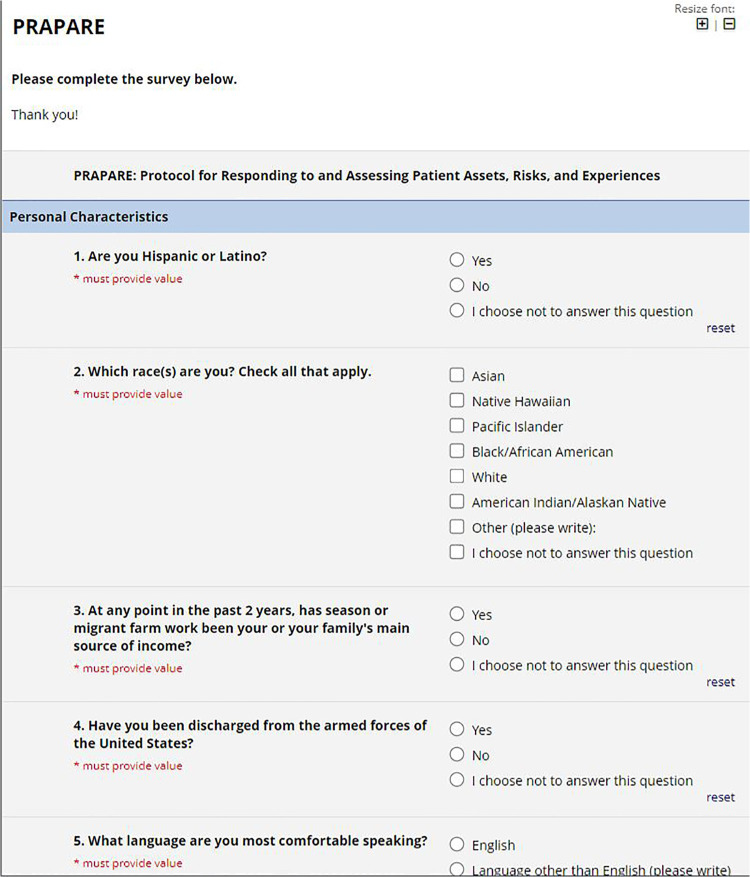
REDCap interface.

REDCap is a widely used traditional web-based tool to collect health and research data in a secure way (25). REDCap can be used to conduct cross-sectional and longitudinal studies, can be integrated with various HIT systems, is customizable, and is free for REDCap consortium partners who have a valid end-user agreement with Vanderbilt University. REDCap is widely used by researchers and providers for clinical data collection (26). Hence, it was chosen as the online form comparison for this study. Figures 1, 2 show screenshots of Dokbot and REDCap, respectively.

We developed a software tool UXEvaluator to conduct the study. UXEvaluator is a web-based tool designed to compare the usability and experience of completing web-based forms or questionnaires. The tool was developed to counterbalance condition sequence across subjects such that each condition was experienced in a random order within-subject. UXEvaluator was used to collect consent and conduct pre- and post-test surveys.

**Figure 3:**
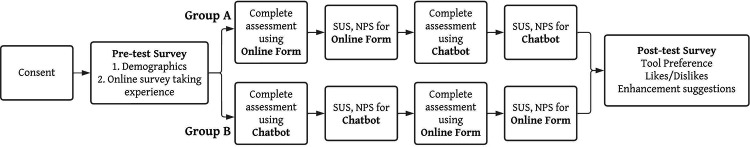
Study design.

Figure 3 summarizes the overall approach of UXEvaluator and this study.

***Step 1.*** *Consent:* Participants were informed about the procedures, benefits and risks, their rights, and provided informed consent prior to the study.
***Step 2.*** *Pre-test questionnaire:* Participants completed a pre-test questionnaire about demographics as described in Section “Study measures”.
***Step 3.*** *Hands-on with Tool 1:* At this step, participants completed the Protocol for Responding to and Assessing Patients' Assets, Risks, and Experiences (PRAPARE) health assessment. We chose this social determinant of health assessment considering its moderate length (21 questions), variety of response formats (e.g., text and numeric inputs, radio buttons, checkboxes), and broad applicability. For the standard PRAPARE question asking for a patient address, participants were instructed to input a fictional address to protect privacy. No personal identifying information was collected. All questions were required. The PRAPARE was administered using either the chatbot (i.e., Dokbot) or the online form (i.e., REDCAP), depending on random group assignment. Group A participants were assigned to complete PRAPARE with the online form first, Group B participants completed the survey using the chatbot first (Step 5).
***Step 4.*** *Tool 1 satisfaction:* Once participants completed the PRAPARE questionnaire using the first randomly assigned tool, they completed SUS and NPS to assess the usability and likelihood to recommend the first tool.
***Step 5.*** *Hands-on with Tool 2:* Users completed the PRAPARE assessment using the second tool. Group A participants completed the assessment using the online form and Group B participants *via* the chatbot.
***Step 6.*** *Tool 2 satisfaction:* Participants completed SUS and NPS for the tool used in Step 5.
***Step 7.*** *Post-test questionnaire:* Participants completed the tool preference measures as described in Section “Study measures”.

### Careless responses and data cleaning

We aimed to recruit a sample of 400 participants on mTurk. However, we observed poor data quality and careless responses with respect to SUS, qualitative responses for open-ended questions on likes and dislikes of each tool, and tool enhancement suggestions. We rejected participants with potential careless responses and recruited 127 additional participants but observed no improvements in data quality. To preserve data integrity and quality, we systematically excluded responses to eliminate acquiescence bias and careless qualitative responses based on previous evidence.

#### Exclusions to eliminate acquiescence bias

Acquiescence biases often refer to participants' tendency to agree or disagree through all questions (27). The SUS is designed with alternating positive and negative statements, such that yea- or nay-saying biases were easily detected with consecutive responses conflicting with one another. It is reported that 8 or more agreements (or disagreements) could suggest that participants could be rushing through the SUS without paying attention (28). We assessed and excluded entries where users chose positive or negative responses (e.g., strongly agree/agree or disagree/strongly disagree) for eight or more statements of SUS. Entries with 8 or more neutral responses (neither agree or disagree) were included in the analysis. We excluded responses if careless responding was observed for SUS related to one (chatbot or online form) or both tools. This resulted in the exclusion of 50 responses. Table 1 shows example responses for this step.

**Table 1:** Examples of participant responses for system usability scale with ≥8 same responses.

SUS 1	SUS 2	SUS 3	SUS 4	SUS 5	SUS 6	SUS 7	SUS 8	SUS 9	SUS 10
Agree	Agree	Agree	Agree	Neither Agree nor Disagree	Agree	Agree	Agree	Agree	Neither Agree nor Disagree
Strongly Disagree	Strongly Disagree	Strongly Disagree	Strongly Disagree	Strongly Disagree	Strongly Disagree	Strongly Disagree	Strongly Disagree	Strongly Disagree	Strongly Disagree
Strongly Agree	Strongly Agree	Strongly Agree	Strongly Agree	Strongly Agree	Strongly Agree	Agree	Strongly Agree	Strongly Agree	Strongly Agree

#### Exclusions based on qualitative feedback

Studies have reported concerns over low data quality and careless qualitative responses from participants recruited on mTurk (29–33). Previously, researchers have noted poor qualitative responses such as short and irrelevant responses, noticeably ungrammatical or nonsense phrases, phrases copied from questions, instructions, online resources, or forms, and text strings repeated among different participants (31, 34). Research suggests removal of potentially fraudulent, careless, and duplicate responses (31).

Four researchers (HS, JI, TO, and HW) developed an exclusion coding scheme based on previous research using four criteria (Table 2). A sample of 72 responses for likes and dislikes of each tool and enhancement suggestions were coded by HS and JI to compute inter-rater agreement. There was a 93% agreement between the two coders, showing high degree of agreement. Coding disagreements resolved by discussion to reach an agreement of 100%. The remaining responses (i.e., *n* = 319) were coded by HS using the updated criteria agreed upon during the resolution of disagreements and reviewed by JI. One of the four exclusion criterion below was assigned to each response:

**Table 2:** Exclusion criteria for qualitative responses.

Exclusion Criteria	Examples			# Excluded
	Chatbot Likes/Dislikes	Online Form Likes/Dislikes	Suggestions	
Unusual Comments	*NICE*	*WELL*	*NICE*	67
Nonsensical sentences	*Try using words that might appear on the page you're looking for. For example, “cake recipes” instead of “how to make a cake."*	*Once you have a REDCap project programmed, data can be imported using … Rate the following ice cream flavors: Hate it. Dislike it. Indiffere nt. Like it. Love*	*YES*	50
Repetitions from website and internet	*Dokbot uses conversation to improve data capture in healthcare. … We can help you quickly create a Dokbot to collect any data you want and store it .Dokbot draws from research since 2015 within the Biomedical Informatics Center (BMIC) at the Medical*	*I would like to know if there are any other Electronica Data Capture (EDC) softwares that … Also, if any of you have used REDCap*	*GOOD*	14
Duplicates comments in all qual responses	*I LIKE THIS*	*SOMAWHAT LIKE*	*GOOD*	4
	*I LIKE THIS*	*SOMEWHAT LIKE*	*GOOD*	

##### Nonsensical sentences

We excluded responses with no meaning (e.g., “*users experience the interaction as the nature I like that and dislike is dislike dokbot are real things”*) or did not relate to the current study (e.g.,*“Try using words that might appear on the page you're looking for. For example, “cake recipes” instead of “how to make a cake”*).

##### Repetitions from website and internet

We also excluded responses which included direct repetitions of statements from product websites, online resources, media coverage or any other publicly available material about the chatbot (such as “*Dokbot uses conversation to improve data capture in healthcare. … We can help you quickly create a Dokbot to collect any data you want and store it .Dokbot draws from research since 2015 within the Biomedical Informatics Center (BMIC) at the Medical”*) or online form (*“The REDCap Consortium, a vast support network of collaborators, is composed of thousands of active institutional partners in over one hundred countries who utilize and support their own individual REDCap systems”*).

##### Unusual comments

We excluded responses with short, unusual comments irrelevant to the question, including responses often written in all capital letters and single words irrelevant responses (e.g., for the question, “what did you like/dislike about 〈tool name〉?,” responses of “NICE”, “good”, and “WELL”). To ensure that valid responses were not eliminated, researchers cross-validated the qualitative comments with their preference of the tool (chatbot or online form). For example, in the questions asking about likes and dislikes of each tool, if a participant responded “VERY LIKE” for the chatbot and “LIKE” for the online form, we reviewed their tool preference. In this scenario, their response was included in analysis if they preferred the chatbot, but it was excluded if they preferred the online form.

##### Duplicates

We removed any duplicate qualitative responses. When we encountered potential duplicate responses, we reviewed the responses for timestamp (studies completed within a short time period), responses to demographic questions (same responses to questions asking age, gender, race, etc.). If the questions contained similar responses, we excluded all potential duplicate responses.

This step resulted in the elimination of 135 responses. Table 2 provides examples of excluded responses and related criteria.

### Data analysis

After applying both the cleaning steps, a total of 206 responses were included in the final consideration for analysis. Quantitative data were tabulated, and descriptive statistics were used to quantify frequency, mean, median, and standard deviation for demographics and pre-test survey experience questions. SUS scores and NPS were calculated based on standardized calculations. Paired *t*-tests and chi-square tests were used to assess differences in SUS, NPS, time taken to complete the PRAPARE assessment, and tool preference. MS Excel and SPSS v28 were used for analyses.

Qualitative responses to open-ended post-test questions were coded to identify likes and dislikes as well as emerging themes related to the tool. Participant responses in full served as the units for coding. Content analysis was used to code participant responses into positive, negative, and neutral categories. Exploratory thematic analysis was completed using MS Excel by one team member followed by thematic analysis using MAXQDA, a qualitative coding platform (35). Over three iterations, a codebook was developed and refined by the entire research team. Another member of the team reviewed the codes, and any discrepancies were resolved through consensus. Themes were quantified and organized by frequency and topic. This process supplemented quantitative analysis. Complex coding query (intersections) was performed to identify what emergent themes were found in juxtaposition to tool opinions.

## Results

### Participant characteristics

Of the 206 participants, 98 participants were in Group A and 108 were in Group B. No significant differences were found in the number of participants in the two groups (χ^2^(1, *N *= 206) = 0.4854, *p *= 0.4860). Table 3 describes the participant characteristics after each data exclusion (elimination of responses with acquiescence bias in SUS and careless qualitative feedback) steps. Table 4 summarizes the groups (A and B) sample size, tool preference, SUS score, NPS score, and time taken to complete the assessment after each exclusion.

**Table 3:** Participant demographics.

The data are reported as *N* (%)	Initial sample *N *= 391	Exclusion 1 *N* = 341	Final Sample *N* = 206
**Age**			
18–30	86 (22.0)	72 (21.1)	51 (24.8)
31–40	171 (43.7)	149 (43.7)	83 (40.4)
41–50	77 (19.7)	71 (20.8)	46 (22.3)
51–60	28 (7.2)	27 (7.9)	16 (7.8)
>60	29 (7.4)	22 (6.5)	10 (4.9)
**Gender**			
Female	128 (32.7)	118 (34.6)	85 (41.3)
Male	262 (67.0)	223 (65.4)	121 (58.7)
Other	1 (0.3)	0 (0)	0 (0)
**Race**			
White	267 (68.3)	230 (67.4)	149 (72.3)
Black or African American	84 (21.5)	78 (22.9)	36 (17.5)
Native American or Alaskan Native	10 (2.6)	7 (2.1)	5 (2.4)
Asian	24 (6.1)	21 (6.2)	11 (5.3)
More than one race	4 (1.0)	4 (1.2)	4 (1.9)
Other	2 (0.5)	1 (0.3)	1 (0.5)
**Ethnicity**			
Hispanic, Latino, or Spanish origin	122 (31.2)	100 (29.3)	30 (14.6)
Not Hispanic/Latino	268 (68.5)	240 (70.4)	175 (85.0)
I don’t know	1 (0.3)	1 (0.3)	1 (0.5)
**Education**			
Pre-school	1 (0.3)	1 (0.3)	0 (0.0)
Middle school (grades 6–8)	2 (0.5)	1 (0.3)	0 (0.0)
Some high school (grades 9–12, no diploma)	3 (0.8)	3 (0.9)	2 (1.0)
High school graduate	14 (3.6)	14 (4.1)	13 (6.3)
Associate degree	16 (4.1)	15 (4.4)	13 (6.3)
Some college (1–4 years, no degree)	28 (7.2)	22 (6.5)	18 (8.7)
Bachelor's degree	229 (58.6)	197 (57.8)	123 (59.7)
Master's degree or higher	98 (25.1)	88 (25.8)	37 (18.0)
**Device used for the study**			
Laptop or Desktop	386 (98.7)	336 (98.5)	201 (97.6)
Smartphone	2 (0.5)	2 (0.6)	2 (1.0)
Tablet	3 (0.8)	3 (0.9)	3 (1.5)
**Familiarity with online surveys**			
Extremely familiar	214 (54.7)	188 (55.1)	132 (64.1)
Moderately familiar	115 (29.4)	97 (28.4)	50 (24.3)
Somewhat familiar	39 (10.0)	38 (11.1)	17 (8.3)
Slightly familiar	19 (4.9)	15 (4.4)	5 (2.4)
Not at all familiar	4 (1.0)	3 (0.9)	2 (1.0)

**Table 4:** Usability and preference characteristics at each exclusion.

		Initial sample *N* = 391	Sample after Exclusion 1 *N* = 341	Final Sample *N* = 206
Group size *n* (%)*	A	196 (50.1%)	171 (50.1%)	98 (47.6%)
	B	195 (49.9%)	170 (49.9%)	108 (52.4%)
	Significance*	χ^2 ^= 0.0026, *p *= 0.9597	χ^2 ^= 0.0029, *p *= 0.9568	χ^2 ^= 0.4854, *p *= 0.4860
Preference *n* (%)*	Chatbot	258 (66.0%)	225 (66.0%)	144 (69.9%)
	Online	133 (34.0%)	116 (34.0%)	62 (30.1%)
	Significance*	χ^2 ^= 39.9616, *p *< 0.001	χ^2 ^= 34.8416, *p *< 0.001	χ^2 ^= 32.6408, *p *< 0.001
SUS m (sd)	Chatbot	62.19 (17.7)	63.73 (18.32)	69.7 (18.9)
	Online	59.83 (16.4)	61.14 (17.13)	67.7 (17.5)
	Significance**	*t* (390) = 3.8005, *p *< 0.001	*t* (340) = 3.6766, *p *< 0.001	*t* (205) = 1.1771, *p *= 0.2405
NPS	Chatbot	24	27	24
	Online	13	15	13
	Significance**	*t* (390) = 4.2514, *p *< 0.001	*t* (340) = 3.8006, *p *< 0.001	*t* (205) = 3.7889, *p *< 0.001
Time in seconds; mean (median, sd)	Chatbot	241.0 (212.0, 126.2)	243.5 (213.0, 129.7)	246.3 (212.5, 139.2)
	Online	164.2 (125.0, 125.5)	167.1 (126.0, 130.9)	168.0 (123.0, 136.2)
	Significance**	*t* (390) = 9.3048, *p *< 0.001	*t* (340) = 8.3224, *p *< 0.001	*t* (205) = 6.3152, *p *< 0.001

* χ^2^ goodness of fit tests with *α* = 0.05.

** Paired *t*-tests, two-tailed, with *α* = 0.05.

### Completion time

The median time to complete the PRAPARE assessment was 89.5 s longer using the chatbot (*median time = *212.5 s) compared to the online form (*median time = *123.0 s). This difference was statistically significant (*t*(205) = 6.3152, *p *< 0.001). Overall, 148 out of 206 (71.8%) participants took longer time (118 s more) to complete the PRAPARE assessment using the chatbot. Most (99.1%; *n *= 107/108) of Group B participants, who completed the PRAPARE assessment using the chatbot first, took longer to complete the assessment using the chatbot. In comparison, 58.2% (*n* = 57/108) of participants who completed the assessment in Group A (online form and then chatbot) took longer to complete the assessment using the online form (median 60 s longer).

### Tool usability and likelihood to recommend

No significant differences were observed in SUS scores between the chatbot (*M* = 69.7) and online form (*M* = 67.7; *t*(205) = 1.1771, *p *= 0.2405). The reported NPS was significantly higher for the chatbot (NPS = 24) compared to the online form (NPS = 13; *t*(205) = 3.7889, *p* < 0.001).

### Tool preference and qualitative assessment of open-ended questions

Of the 206 participants included in the analyses, 144 (69.9%) preferred the chatbot and 62 (30.1%) preferred the online form. There was a statistically significant difference in the preference for the chatbot over the online form (χ^2^(1, *N *= 206) = 32.6408, *p *< 0.001).

The post-test questionnaire asked participants about their likes and dislikes towards the user tool and enhancement suggestions. The qualitative responses were thematically analyzed to identify common emerging themes. Table 5 presents the common emerging themes and example responses related to both the tools.

**Table 5:** Common emerging themes and examples.

	Codes	*N*	Definitions	Examples
User Descriptor	Intuitive	131	Describing platform as easy to use, comfortable, or natural	*Since it was presented as a chat session, it had a friendly vibe to it. Was easy to stay on task.*
	Interactive/Engaging	72	Describing how platform increases user interest, captivates the user, or involves them in the process of filling out survey	*Seems personal, easy to use, and more engaging.*
	Standard	60	Describing platform as expected, old-school, or average--often times, something previously experienced	*It was more what you would expect from a survey system. It was easy to use too.*
	Quick	35	Describing platform as taking a short or shorter amount of time to navigate	*I liked that I could use the survey at my own pace. I was able to quickly answer questions without having to wait for a prompt.*
	Time Consuming	21	Describing platform as taking a long or longer amount of time to navigate	*It felt interactive but took longer.*
	Unfamiliar	18	Describing platform as confusing due to novelty or peculiar	*The font and format seemed outdated and unnatural.*
Interface Quality	Interface	74	Specific mention of the formatting of the platform	*I loved the layout and it seemed like I was having a discussion with a bot. This made answering and using dokbot more fun and interactive.*
	Navigation	62	Specific mention to how questions within survey are displayed or presented during use of the platform	*REDCap has the scrolling thing which takes up more space and is a little harder to move forward.*
	Formal	8	Describing that the format of the platform felt professional	*It was too formal. It felt like I was reading and filling out a government document.*
	Security Issue	7	Note of a potential security risk or feelings of invasiveness due to platform type or question	*I just felt less secure using a chat function.*
	Informal	5	Describing that the format of the platform felt informal or unprofessional	*I did not like the “chat” format. It seems too informal for a health questionnaire.*
	Integrated	3	Specific mention of the interface format being set up well in terms of coordination with purpose of survey	*It's much easier for consumer to respond to question, and the interface is more refined and integrated.*

Participants often perceived the chatbot's interface as conversational, interactive, intuitive–comparable to having a natural conversation. One participant said, “*I did like the way it was designed and had the feeling that I was talking to a human.”*

Participants also felt the chatbot was easy (intuitive) to use. One participant mentioned, “*[chatbot] was very easy to use and it was amazing to handle,”* and another participant commented, “*I liked how easy it was to use [chatbot]. I liked how it seemed as if it were progressing through questions without me having to click things. I didn't dislike anything about [chatbot]”*.

Participants also commented on the design and layout of the chatbot's interface. Participants perceived that the interface was modern and easy to navigate. Considering the mobile-first approach, participants appreciated that they did not need to scroll through the screen. Participants also liked the presentation of the information and the systematic flow. As the chatbot presents questions one-by-one, there are fewer chances of skipping a response and focusing on the presented question or task. A participant commented, “*it was easier to fill out without worrying if you accidentally skipped a question.”* However, some participants perceived that the display was small, presented a large amount of information, and felt informal for healthcare assessments.

Some participants also reported the chatbot felt time-consuming, which aligned with the longer average completion time for the chatbot. They mentioned that “*it's a bit slower compared to seeing all questions at once on the [online] survey.”* Whereas most participants perceived the chatbot as natural and engaging as talking to a human, a few participants were concerned about the privacy of the information due to the chat-based nature. One participant noted, “*I just felt less secure using a chat function.”*

When discussing interface quality, six participants mentioned security issues with one participant mentioning security twice in their responses. One participant commented that the online form was a “*simple and honest way to gather information. No manipulation or misleading*.” While three participants specifically noted some type of discomfort as associated with the chat function (Table 5), the other three participants felt the chatbot was asking sensitive questions: “*Seemed a bit invasive. Not sure what the actual purpose was. Why would it need my address?*” and “*It seemed to ask really personal questions and it made me suspicious…*”

Participants often perceived the online form's interface to be less favorable. Although some participants commented that the interface was simple, straightforward, formal, and presented all information at once, participants commonly perceived the interface as old school or traditional. Participants disliked the design and layout of the assessment including, design and size of the response areas, alignment of questions, color scheme, and readability. One participant commented, “*it's kinda stressful with the way the items are aligned.”*

Participants did acknowledge that the online form's interface was quick and less time-consuming compared to the chatbot, but it was also less engaging and efficient: “*I felt [the online form] was a bit faster. However, I felt it was less personable than the other [chatbot] tool”*.

Additionally, complex coding queries were done with the most populated codes to determine whether there was discussion of multiple topics by participants in a meaningful way (Table 6). *Navigation* was often discussed in tandem with whether the product was *intuitive* (25 codes), *quick* (13 codes), and *interactive/engaging* (10 codes): “*I thought it was exceedingly intuitive, snappy, and it felt more personable and engaging than REDCap. I liked the chatbot nature of dokbot. It allowed for much more efficient progression through the questions. There wasn’t anything I disliked.*”

**Table 6:** Positive and negative perception resulting from complex coding query.

	Platform	
Type of Comments	Chatbot (*N*)	Online Form (*N*)
Positive	168	113
Negative	39	86
Neutral	8	18
Positive and negative	10	15
**Chatbot**		
**Code**	**Positive (*N*)**	**Negative (*N*)**
Standard	0	1
Time-consuming	5	12
Quick	18	0
Interactive/engaging	59	6
Intuitive	81	4
Unfamiliar	0	8
Navigation	29	8
Interface	30	9
Integrated	2	0
Informal	1	4
Formal	1	0
Security issue	2	6
**Online Form**		
**Code**	**Positive**	**Negative**
Standard	20	37
Time-consuming	2	6
Quick	12	0
Interactive/engaging	59	6
Intuitive	44	9
Unfamiliar	2	7
Navigation	15	16
Interface	17	22
Integrated	0	1
Informal	0	0
Formal	3	3
Security issue	1	0

Meanwhile, *interface* was most often discussed in tandem with *interactive/engaging* (10 codes), *intuitive* (15 codes)*,* and *unfamiliar* (4 codes): “*I just didn’t feel I was able to read and interact with it in a comfortable manner…and it felt a bit more rushed as if someone was waiting on me to answer the question…*”

When looking at whether participants provided *positive, negative,* or *neutral* commentary, the chatbot received majority positive responses (59.8%), while the online form received majority negative responses (68.8%). When further analyzed, the chatbot received most of its positive comments regarding its *interactive/engaging* and *intuitive* interface: “*It seems like chatting with a real human, it is so engaging*.” The online form, on the other hand, received most of its positive commentary regarding its *standard* and *intuitive* interface: “*It was more what you would expect from a survey system. It was easy to use too*.” Further, the online form's negative comments focused on that same *standard* trait: “*Bland. straightforward.”*

## Discussion

### Main findings

Our goal was to explore virtual conversational agents or chatbots as an effective means to facilitate healthcare data collection. We present one of the earliest studies assessing user preferences of chatbots for data collection in comparison with traditional online forms. Participants preferred the chatbot's interface, reporting higher usability and significantly higher likelihood to recommend it as a data collection tool. The reported SUS scores for the chatbot (69.7) was higher than the average industry benchmark score of 68 (19, 36). The NPS score of the chatbot (NPS = 24) was higher than the online form (NPS = 13). The chatbot received the majority (59.8%) of positive responses with comments remarking on its conversational, intuitive nature, “*…it seemed like I was having a discussion with a bot. This made answering and using dokbot more fun and interactive.”* While the standard online form's interface received the majority (68.8%) of negative responses, some of its most positive participant comments praised its familiarity: “*Very standard, easy to use and worked well, I could get a larger overview of the entire survey*.” One participant encapsulated the feeling of the form being standard simply, “*Bland. straightforward*.” The modern layout and the mobile, human-like interface and design of the chatbot was the source of the most positive comments. Indeed, though the standard interface survey was considered intuitive (most likely due to prior exposure), the chatbot was intuitive due to its engaging nature: “*Since it was presented as a chat session, it had a friendly vibe to it. Was easy to stay on task.”* Even with negative comments, the chatbot appeared to make data collection easier and more enjoyable to navigate for participants, suggesting chatbot could be a better way to collect data.

The assessment took significantly longer (i.e., 89.5 s more) to complete using the chatbot compared to the traditional online form. This could be due to the conversational format of the chatbot, which presents questions and information in a controlled sequence rather than presenting questions all at once. This could lead to increased scrolling to previously answered questions and more clicks while responding to questions and other presented information, ultimately leading to a longer time to complete the assessment. Qualitative analyses supported these results, showing that while some (*n *= 12/206) participants perceived the chatbot as time consuming, an equal number of participants (*n *= 12/206) noted the online form's interface felt faster: “*The layout appeared simple and old-school. But I found it to be easier and faster to work on than the dokbot*.” However, only 5.8% (*n *= 12/206) of participants reported chatbots felt time-consuming in their qualitative responses, so it is possible that the chatbot was more engaging and not perceived as taking longer to complete; one participant noted, “*It seemed to move a lot quicker and less tedious*” and another that “*it felt interactive but took longer*.” Similar findings have been attributed to engagement in previous research (11). More research focusing on the user behaviors while interacting with the chatbot and the quality of data collected could help better understand the equilibrium between experience with the chatbot and impact of completion time.

While the chatbot may have taken longer to complete than the online form, time to complete was not necessarily a negative consideration. The chatbot was reported to be faster to complete by 18 (8.7%) participants, while the online form was reported as faster by 12 (5.8%). Results show that the chatbot was still preferred, even with greater time consumption, suggesting substantial engagement and an intuitive interface have a greater weight on preferences. One participant noted this interplay, stating, “*I think time seems to go faster with dokbot. It has the feel of a more personal conversation, instead of the basic form survey…*” While prior research has shown that chatbots increase feelings of engagement, this study also shows engagement may supersede the ideal of quick healthcare forms (37). Consequently, patients may spend more time providing quality information to their healthcare provider. Further research with a clinical sample size is needed to determine quality and patient preference on this topic.

In addition, a few participants showed concerns for sharing personal health information *via* chatbot. It is possible that the lack of familiarity with the chatbot as a tool for health data collection or a third-party solution could have raised these concerns. While three participants mentioned their security concern using a chatbot, the other three instances of security concerns may be a result of participants completing a health survey in a study environment. Indeed, one of these participants felt uncomfortable, asking “*…why would it need my address?*”, though such information is typical in healthcare assessment forms. Additionally, mTurk educates participants and researchers not to provide specific location information, so it is possible that some participants did not notice the instructions to input a fictional address provided by the researchers (38). Ultimately, reassuring participants and communicating compliance with privacy regulations such as HIPAA and data safety could potentially alleviate these concerns. Though this study had few cases of security concerns emerge (2.9%, *n *= 6/206), future research should explore patients' feelings of security and appropriateness of chatbots in collecting their health data for their health care provider.

A major cause for concern in this study was the quality of the data collected *via* mTurk. Research has shown that careless, nonsystematic, and potential bot responses have resulted in removal of a significant amount of data in some studies (30, 31). Although our larger sample size without excluding any participants echoed current findings (chatbot preferred over online form), we observed careless and missing quantitative and qualitative responses resulting in poor data quality. To improve data quality and preserve integrity, we performed a data cleaning process informed by literature and research. These exclusions resulted in an 47.3% reduction in our sample size (i.e., 206/391). In future, researchers should consider these factors and establish more robust screening approaches and strategies to identify and avoid careless and bot responses.

Overall, our study findings establish chatbots as a modern, friendly, and intuitive approach to health data collection compared to traditional online forms or the conventional use of chatbots as therapy or education delivery mechanisms in healthcare. With the growing use of mobile devices to obtain health information, this approach can help collect high-quality, complete, more accurate data from patients, thereby enhancing the processes and workflows of health data collection (39, 40).

### Limitations

We recruited participants *via* mTurk, which may not be representative of the general population. We aimed to recruit participants who were 18 years or older but made no further specifications for age, gender, race, or education. However, our findings were consistent with previous studies with well-educated and underserved populations (11–14).

Considering the remote and unmoderated nature of this recruitment, individuals may have completed the assessments and questionnaires inaccurately or disingenuously. It is possible that individuals may have completed the assessment in a hurry or taken additional time to complete the assessment at their own convenience. In the future, researchers should ensure more objective checks and criteria for approval of mTurk responses and refer to previous research on the challenges of recruitment *via* public platforms such as mTurk.

Further, biases can occur from paid survey pools, specifically at low levels of compensation (41). It is possible that this study may see framing effect bias due to this recruitment approach; however, such a framing effect is more likely seen regarding questions of money and risk–topics not considered in this study.

We targeted a large-scale recruitment of 400 individuals, but this approach resulted in inclusion of only 206 individuals. The loss of statistical power could impact the generalizability of these results.

Lastly, it is possible that the preference of chatbot could be related to its conversational nature, but also could be due to other factors such as its visual and structural information representation. In this study, the chatbot and online form differed in their visual appearance and information representation. The chatbot presented a modern interface with systematic one-by-one questions compared to the online form, which presented all questions at once. The chatbot also included features such as progress tracking which were not present in the online form. Hence, the findings may not be generalizable in comparison to other modern online forms presenting one-by-one flow of questions. Future studies should compare Dokbot with other HIPAA-compliant healthcare tools designed for online clinical and research data collection.

### Future work

This is one of the first studies assessing individual preferences towards two different tools for health data collection. Our findings have identified the areas of improvement for Dokbot, including completion time, information, and conversation presentation, as well as privacy and security concerns. We aim to conduct future studies to better understand these concerns and identify ways to improve Dokbot. Also, this study focused on the usability and experience of collecting data using two different interfaces: a chatbot and an online form. We did not look into the quality of data collected using these forms. Future studies should focus on assessing the data quality, including the accuracy, reliability, and completeness of health data.

Though we aimed to conduct the study with 400 participants, we have reported partial results including 206 participants. In the future, we aim to identify a better recruitment platform and conduct large scale validation of our findings. In addition, we are conducting a detailed, moderated study comparing preferences of the chatbot with online form as well as paper form. This study aims to better understand preferences with the inclusion of validated measures of perceived usefulness and ease-of-use based on the Technology Acceptance Model, cognitive and task load based on the NASA Task Load Index (NASA-TLX), and system satisfaction using the IBM Computer System Usability Questionnaire (CSUQ) (42–44).

Our study did not include many older adults (age >60 years; *n *= 10, 4.9%). Older adults tend to experience greater difficulty navigating online interfaces, and little research has explored their unique experiences and challenges with online health data collection (12). We are in the process of systematically reviewing the literature about chatbots for health data collection among older adults and evaluating the cognitive load, usability, and ease-of-use of chatbot-delivered health forms among 300 older adults using the platform Prolific.co (45). We chose this online platform as researchers have reported on the high quality of data collected using Prolific.co (29).

Collectively, the outcomes of these studies will help develop strategies and recommendations for improving Dokbot as a more intuitive and interactive healthcare data collection tool.

## Conclusion

This manuscript presents an early study assessing the experience and preference of chatbots as health-data capture mechanisms. The findings demonstrate chatbots can be an intuitive and useful approach to modern healthcare data collection, increasing engagement and interaction among patients. Factors negatively influencing application of chatbots in data collection may include increased completion time and privacy concerns. However, our findings demonstrated that high engagement, intuitiveness, and interactive experience supersede the negative influences. The findings of this research will inform design and development of recommendations and best practices for chatbots for healthcare data collection.

## Data Availability

The raw data supporting the conclusions of this article will be made available by the authors, without undue reservation.
